# Stability of dosiomic features against variations in dose calculation: An analysis based on a cohort of prostate external beam radiotherapy patients

**DOI:** 10.1002/acm2.13904

**Published:** 2023-01-11

**Authors:** Lingyue Sun, Wendy Smith, Charles Kirkby

**Affiliations:** ^1^ Department of Physics and Astronomy University of Calgary Calgary Alberta Canada; ^2^ Department of Medical Physics Tom Baker Cancer Centre Calgary Alberta Canada; ^3^ Department of Oncology University of Calgary Calgary Alberta Canada; ^4^ Department of Medical Physics Jack Ady Cancer Centre Lethbridge Alberta Canada

**Keywords:** dose calculation, dosiomics, stability

## Abstract

**Introduction:**

Interest in using higher order features of the planned 3D dose distributions (i.e., dosiomics) to predict radiotherapy outcomes is growing. This is driving many retrospective studies where historical data are mined to train machine learning models; however, recent decades have seen considerable advances in dose calculation that could have a direct impact on the dosiomic features such studies seek to extract. Is it necessary to recalculate planned dose distributions using a common algorithm if retrospective datasets from different institutions are included? Does a change in dose calculation grid size part way through a retrospective cohort, introduce bias in the extracted dosiomic features? The purpose of this study is to assess the stability of dosiomic features against variations in three factors: the dose calculation algorithm type, version, and dose grid size.

**Methods:**

Dose distributions for 27 prostate patients who received EBRT were recalculated in the Eclipse Treatment Planning System (Varian Medical Systems, Palo Alto, California, USA) using two algorithms (AAA and Acuros XB), two versions (version 13.6 and 15.6), and three dose grids (2, 2.5 s, and 3 mm) – 12 dose distributions for each patient. Ninety‐three dosiomic features were extracted from each dose distribution and each of the following regions‐of‐interest: high dose PTV (PTV_High), 1 cm rind around PTV_High (PTV_Ring), low dose PTV (PTV_Low), rectum, and bladder using PyRadiomics. The coefficient of variation (CV) was calculated for each dosiomic feature. Hierarchical clustering was used to group features with high and low variability. Three‐way repeated measures ANOVA was performed to investigate the effect of the three different factors on dosiomic features that were classified with high variation. Additionally, CVs were calculated for cumulative dose volume histograms (DVHs) to test their ability to detect the variations in dose distributions.

**Results:**

For PTV_Ring, PTV_Low, and rectum, all the dosiomic features had low CV (average CV *≤* 0.26) across the varying dose calculation conditions. For PTV_High, six dosiomic features showed CV > 0.26, and dose calculation algorithm type and grid size were the major sources of within‐patient variation. For bladder, one dosiomic feature had average CV > 0.26, but none of the three dose calculation‐related factors led to a statistically significant variation. The CVs for all the DVHs were very small (CV < 0.05).

**Conclusion:**

For all the regions‐of‐interest examined in this study, the majority of the dosiomic features were stable against variations in dose calculation; however, some of the dosiomic features for PTV_High and bladder had significant variations due to differences in dose calculation details. DVHs were detecting less variation than dosiomic features.

## 1 INTRODUCTION

Dosiomics is an emerging field that aims to extract high‐dimensional minable data from 3D radiation dose distributions and analyze that data to assist clinical decision‐making.^1^, ^2^, ^3^, ^4^, ^5^ Drawing from principles developed in the field of radiomics, dosiomics refers to the extraction of shape features, first‐order statistical features, and texture features that represent the statistical relationships between the neighboring voxels within a defined region of interest (ROI). Because they incorporate spatial information, including dosiomic features in outcome prediction models is likely to have better performance than models based exclusively on dose‐volume histograms (DVHs).^2^, ^6^ There is increasing interest in using dosiomics as a tool in radiation therapy for normal tissue complication probability modeling,^7^ radiation‐induced toxicity prediction,^1^, ^2^, ^6^ and tumor control outcome prediction.^3^, ^4^, ^5^

The construction of machine learning based outcome prediction models requires a large amount of data. To collect enough to generate models that are statistically meaningful, investigators will frequently turn to available collections of historical data for retrospective analysis or data across many different centers. But the technology used in radiation therapy dose calculation has changed considerably over the past decades due to advances in computing power, speed, and connectivity.^8^, ^9^, ^10^, ^11^ Further, different centers may use different algorithms or dose calculation parameters.^12^, ^13^, ^14^, ^15^ In turn, these differences may introduce variations in the higher order dosiomics patterns from center‐to‐center or through time,^16^, ^17^ and ultimately reduce the efficacy of the outcome prediction models based on such data.

For predictive models to generate optimal and reproducible results, the input features need to be stable. In the context of using dosiomics for radiation therapy outcome prediction, this means the dosiomic features should have minimal variation as dose calculation parameters vary. These parameters include dose calculation algorithm type, version, and dose grid size. In this work we seek to systematically quantify such variations in the context of prostate cancer radiotherapy plans so as to identify situations in which recalculation of dose distributions is warranted in order to optimize the efficacy of a given model.

In this work, we build on a few studies that have attempted to investigate this topic. In one study, the stability of dosiomic features with variations in grid resolution and algorithm was studied using 18 clinically delivered dose distributions from different tumor sites.^17^ The authors found that variations in dose grid resolution and algorithm had a significant impact on some of the PTV's gray level co‐occurrence matrix features.^17^ However, with a minimum of one patient included for each tumor site, results may not be very robust. Because different imaging protocols would have been used for those patients, it would be hard to rule out the influence of imaging‐related factors. Additionally, reproducibility and stability of different features may be tumor site‐specific or patient anatomy‐specific. In another study, reproducibility, stability, and sensitivity of dosiomic features against multiple technologies, techniques, treatment planning systems were investigated using dose distributions generated for a simplified head and neck phantom with different dose calculation algorithm types and dose grid in a multicenter setting.^16^ In this study, only 63.2% of the PTV's dosiomics features were found to be stable and reproducible and the percentage was even lower for the left and right parotids.^16^ While this study is valuable, the results may not be generalized to a real patient cohort due to anatomical differences between a phantom and a real patient and the anatomical variations between patients.

We aim to identify the features that are stable and features that are not stable against common differences in approaches to dose calculation and investigate the variable(s) that can have a significant impact on the non‐stable features. This work helps to identify the dosiomic features that can be included when investigating the prognostic ability of dosiomic features in predicting post‐EBRT tumor control outcome and radiation‐induced toxicity outcome despite inconsistency in dose calculations. In this study, we first characterize the variations of a set of common dosiomic features as parameters of interest for 27 prostate EBRT patients, then, statistical tests are applied to examine the statistically significant contributing factor(s) for those highly variable dosiomic features. We will examine the statistical impact of the dose calculation variations on dosiomic features with the assumption that future dosiomics‐outcomes investigations will likely rely on statistically resolvable differences in these features to identify correlations with various outcomes. The clinical impact of variations in dose calculation is beyond the scope of this study. We also quantified the variations in cumulative dose volume histograms (DVHs) and assessed its ability in detecting variations in dose distributions.

## 2 MATERIALS AND METHODS

### 2.1 Dose distribution dataset

Twenty‐seven prostate cancer patients who had planning CT scans acquired using the same imaging protocol and treated with two‐phase sequential boost volumetric modulated arc therapy (VMAT) at our center between September 2014 and August 2017 were retrospectively selected. The planning CT image sets were acquired on one of the two Philips Brilliance Big Bore CT scanners (Philips Medical Systems, Cleveland, Ohio, USA). The two CT scanners that had the same electron density calibration curves for Hounsfield unit ≤ 17,742.5. None of the patients had metal implants. All the planning CTs had a slice thickness of 3 mm with 120 kVp and 400 mAs. The axial plane had a matrix of 512 × 512 with pixel spacing in the range of 0.94–1.17 mm. In EBRT phase one, prostate, seminal vesicles (SVs), and elective pelvic lymph nodes with a 10 mm margin (PTV_Low) were prescribed with 4500–4680cGy in 23–26 fractions; In phase two, a boosting dose of 2800–3240 cGy in 14–18 fractions was delivered to the prostate ± SVs with a 7–10 mm margin (PTV_High). The plans were created in the Eclipse Treatment Planning System (Varian Medical Systems, Palo Alto, California, USA) on a Varian TrueBeam linac using volumetric modulated arc therapy and were clinically delivered for those patients. For a given patient, each of the two clinical plans (phase one and phase two) was copied and the dose was recalculated with different dose calculation algorithm types, versions, and dose grids as shown in Table 1 using Eclipse scripting. The DICOM datasets for dose distributions and structure set for each patient were exported.

**TABLE 1:** Dose calculation algorithm (DCA) types, versions, and dose grid used in this study

DCA types	DCA versions	Dose grids
AAA (dose to water)	13.6	2 mm
		2.5 mm
		3 mm
	15.6	2 mm
		2.5 mm
		3 mm
AXB (dose to medium)	13.6	2 mm
		2.5 mm
		3 mm
	15.6	2 mm
		2.5 mm
		3 mm

While it is challenging to estimate what specific effects varying these parameters will have on the dosiomic features, they are each reasonable to investigate closely. For example, with a larger dose grid, more volume averaging will occur. In turn, this can decrease dose gradients on a voxel‐to‐voxel scale and potentially change quantities associated with the dosiomic texture. Currently, at our institution a dose grid size of 2.5 mm is used clinically for all the prostate EBRT plans, and we examined dose grid sizes of 2, 2.5, and 3 mm in this study. Differences in dose calculation algorithm types AcurosXB (AXB), a grid‐based Boltzmann‐solving algorithm, and the Anisotropic Analytical Algorithm (AAA), both from Varian Medical Systems (Palo Alto, California, USA) are well documented.^18^, ^19^, ^20^, ^21^, ^22^ For example, AXB estimates a lower and more accurate target dose than AAA in low‐density media (e.g., lung)^19^, ^21^, ^23^ and at air/tissue interfaces.^20^, ^22^ While upgrading system versions over time, generally speaking, the fundamental algorithms have remained the same; however, customer release documentation details numerous minor bug fixes and feature additions. In version 15 for example, AXB calculations include an option to perform calculations using a graphics processing unit rather than the central processing unit.^24^ Maximum CT number values in the CT to relative electron density or mass density calibration curves were extended to 29,768 HU, while for version 13, the maximum HU was 10,000.^24^ While one may not expect such changes to yield statistically different results in dosiomic features within typical prostate plans, as small changes accumulate, it becomes prudent to evaluate this explicitly.

### 2.2 Dosiomic feature extraction

All the patients’ DICOM RT files were first anonymized. A binary mask for each ROI (PTV_High, PTV_Low, rectum, bladder, and PTV_Ring = 1 cm rind around PTV_High) was created from the DICOM structure set using the python package ‘DicomRTTool’.^25^ Those ROIs were chosen because they were commonly used for investigating the relationship between their planned dose and tumor control/toxicity outcomes.^1^, ^4^, ^26^, ^27^, ^28^ The binary masks were all resampled to be 1mm×1mm×1mmusing nearest neighbor interpolation to preserve the binary label value. A binary mask defines the ROI on the 3D dose distribution and the dosiomic features were extracted within the defined ROI. For each patient, the dose distributions for the two phases were summed. As a result, each patient had a total of 12 plan sum dose distributions covering the various dose calculation grid sizes, versions, and types as shown in Table 1. Then, the plan sum dose distributions were resampled onto a finer 1mm×1mm×1mmresolution using B‐spline interpolation for analysis. The dose distributions had voxel gray level values that correspond to the planned absolute dose in the unit of Gray. For each dose distribution, dosiomic features were extracted from the ROI using PyRadiomics in Python with absolute dose levels binned into 100 discrete levels from minimum to the maximum dose of the ROI.^29^ The extracted features included first‐order statistics features (18 features), gray level co‐occurrence matrix features (24 GLCM features), gray level dependency matrix features (14 GLDM features), gray level run length matrix features (16 GLRLM features), gray level size zone matrix features (16 GLSZM features), and neighboring gray‐tone difference matrix features (5 NGTDM features). A brief description of each gray level matrices can be found in the supplementary Table S1 and the complete list of features is listed in supplementary Table S2. All of these dosiomic features have been used by other studies investigating their relationship with tumor control or toxicity outcomes.^1^, ^2^, ^3^, ^4^, ^5^, ^6^, ^7^

### 2.3 Data analysis

In our study, to evaluate the variability of the dosiomic features due to variations in dose calculation, for each ROI, the coefficients of variation (CVs) were calculated for each dosiomic feature based on the 12 dose distributions per patient. The CV evaluates the extent of variability of the dosiomic feature relative to its mean value. It is defined as: $CV\;=\;\frac{\sigma }{\left|\mu \right|}$, where σ is the standard deviation and μ is the mean of a dosiomic feature. It has been used in the context of assessing reproducibility and stability of radiomics features^30^ and dosiomic features.^16^ In this study, a similar analysis was performed for cumulative DVHs to test their ability to detect the variability in dose distributions. DVH was discretized into D1%[%], D2%[%], …, D100%[%], and CV was calculated for each of these.

For all the ROIs, the variations of dosiomic features’ CVs were examined using heatmaps. For the ROI that had the greatest variation of CVs across all dosiomic features, hierarchical clustering using Euclidean distance and average linkage was performed. The purpose was to group features into two clusters, one cluster of features with minimal variation and the other cluster of features with larger variation. This helped to decide a threshold value between stable and unstable dosiomic features. For the group of features with minimal variations, we averaged the CVs for each dosiomic feature across all patients (average CV). We found the feature with the maximum “average CV.” We chose this value as the threshold for further data analysis. If a feature's average CV was above this threshold, the feature was considered unstable and vice versa. Similar hierarchical clustering was also performed for grouping patients and investigating if the clustering was related to the CT scanner used.

**FIGURE 1:**
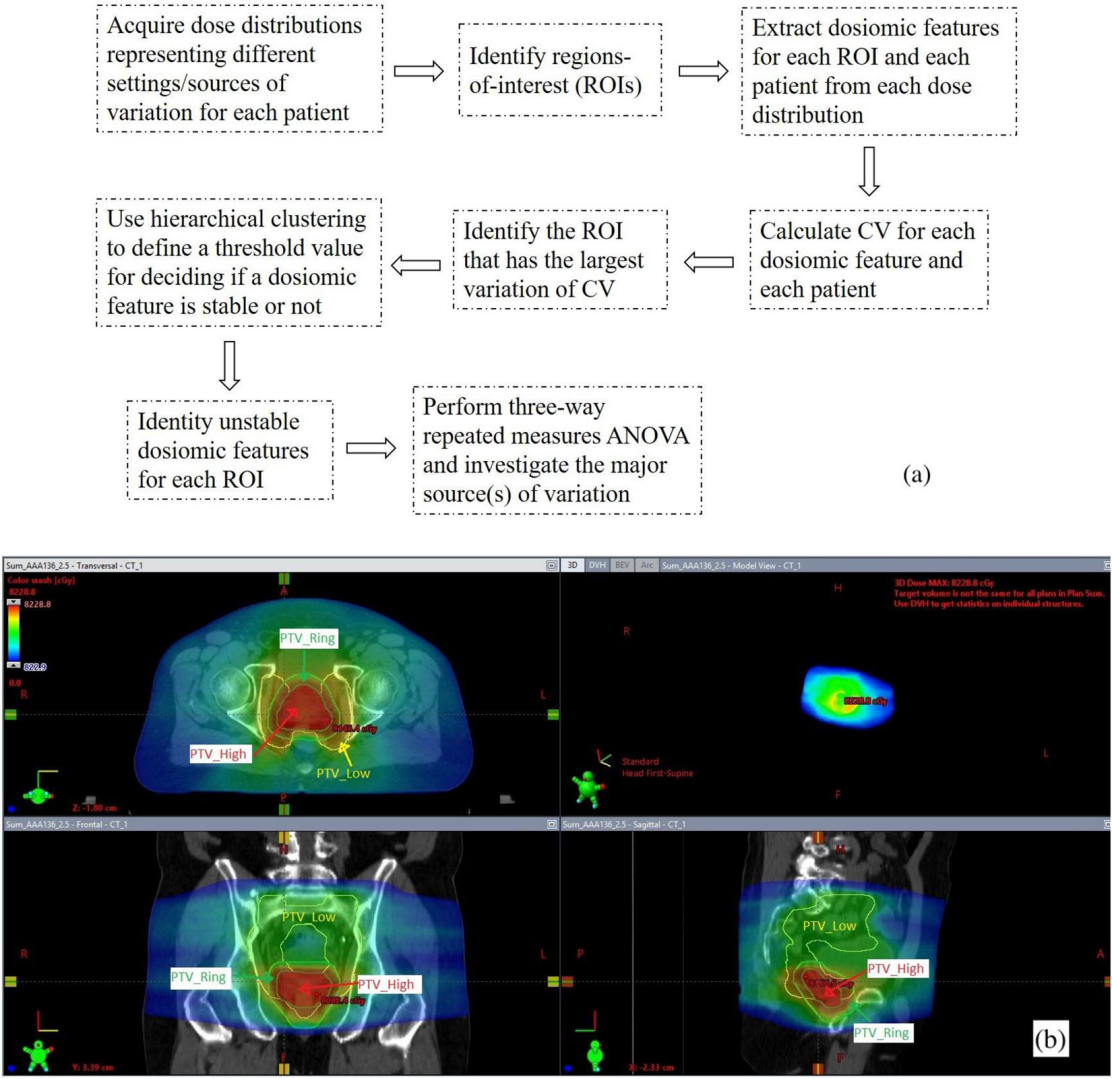
(a) Workflow in this study. (b) Examples of the ROIs used in this study

The unstable features were further analyzed to investigate the major source(s) of variation (dose calculation algorithm, version, and dose grid size). For dosiomic features that were not normally distributed, the values were first converted to a logarithm form. The normality assumption was checked using a Shapiro test for each unstable feature.^31^ Three‐way repeated measures analysis of variance (ANOVA) was used to simultaneously investigate the effect of the three independent variables on each of the unstable dosiomic features by comparing the differences in group means.^32^ Outliers were removed for analysis because three‐way repeated measures ANOVA is very sensitive to outliers. Mauchly's test was used for checking sphericity^33^ and if the independent variable failed the sphericity assumption, a Greenhouse‐Geisser correction was applied for three‐way repeated measures ANOVA. The Bonferroni correction^34^ was used to account for the repeated statistical testing as three‐way repeated measures ANOVA was repeatedly used for all unstable features. According to this correction, a p‐value < $\frac{0.05}{number\;of\;unstable\;features}$ was considered statistically significant. All the analyses in this study were performed in R (version 4.0.3, R Foundation for Statistical Computing, Vienna, Austria). The workflow in this study is summarized in Figure 1.

## 3 RESULTS

**FIGURE 2:**
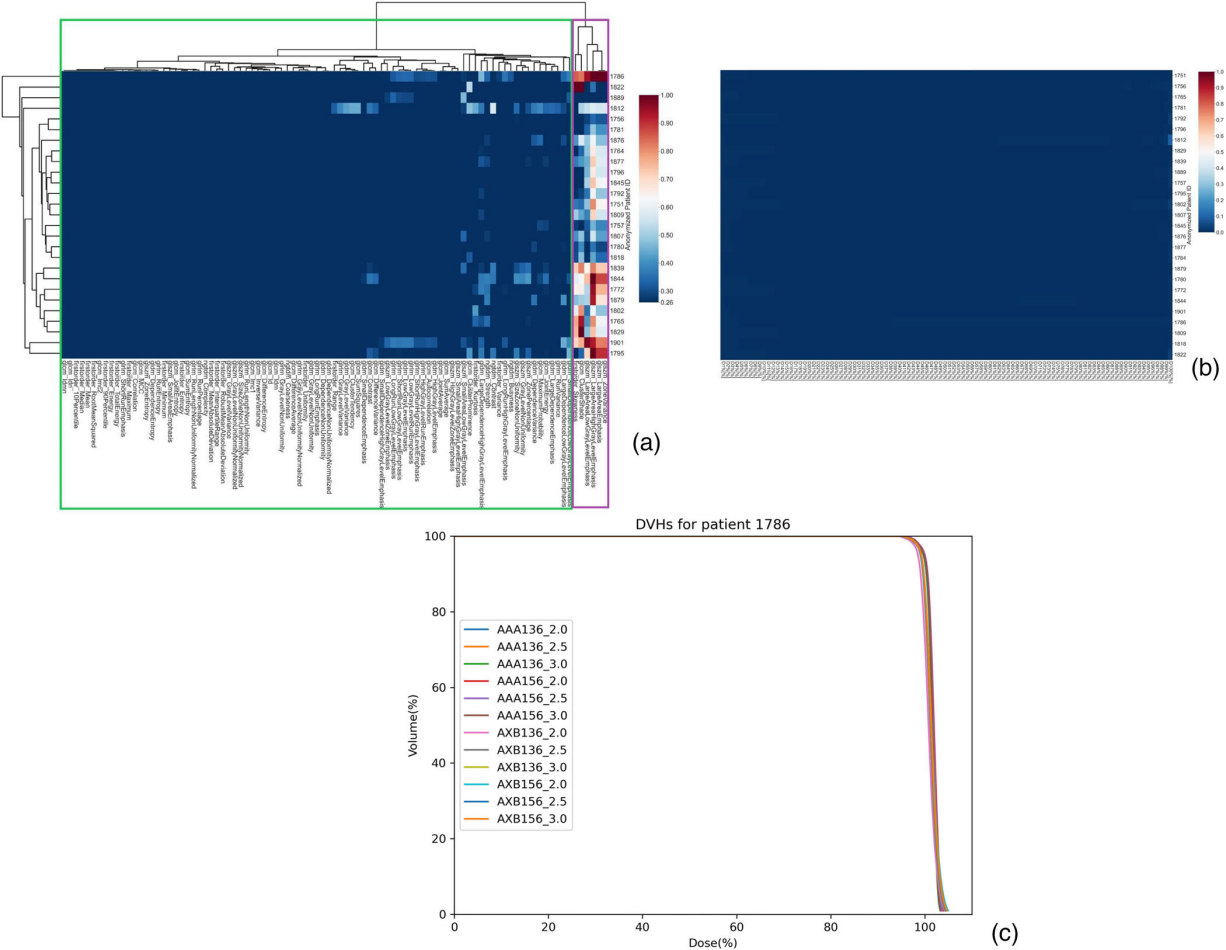
Heatmaps showing the coefficient of variation for dosiomic features (a) and DVHs (b) of PTV_High. A dark red cell for a dosiomic feature/dosimetric parameter and a patient means this particular dosiomic feature/dosimetric parameter has a large amount of variation when dose distributions were calculated using different dose calculation algorithm type, version, and dose grid. In the contrary, a dark blue cell means minimal variation, and thus the dosiomic feature/dosimetric parameter is stable against variations in dose calculation details. The dendrograms of figure (a) showed hierarchical clustering of dosiomic features and patients. Purple rectangle: the features that had large CVs thus not stable against variations in dose calculation. The green rectangle includes features that had small CVs and thus were stable against variations in dose calculation (maximum average CV: 0.26). DVHs had minimum CVs and did not detect much variation in dose distribution. Figure (c) shows the DVHs for the patient 1786 who had the greatest variations in the unstable features

**FIGURE 3:**
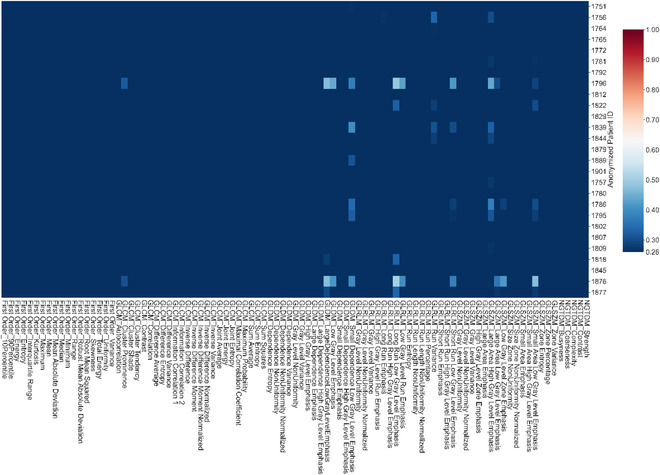
Heatmap showing the coefficient of variation for dosiomic features of PTV_Low. There were minimal variations (average CV≤0.26) for all the dosiomic feature—stable against variations in dose calculation

**FIGURE 4:**
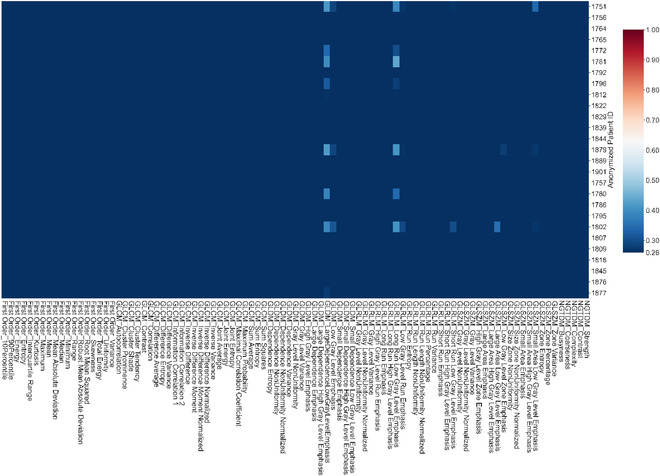
Heatmaps showing the coefficient of variation for dosiomic of PTV_Ring. PTV_Ring = 1 cm ring around PTV_High, the region that had the greatest dose fall off. There were minimal variations (average CV≤0.26) for all the dosiomic features—stable against variations in dose calculation

**FIGURE 5:**
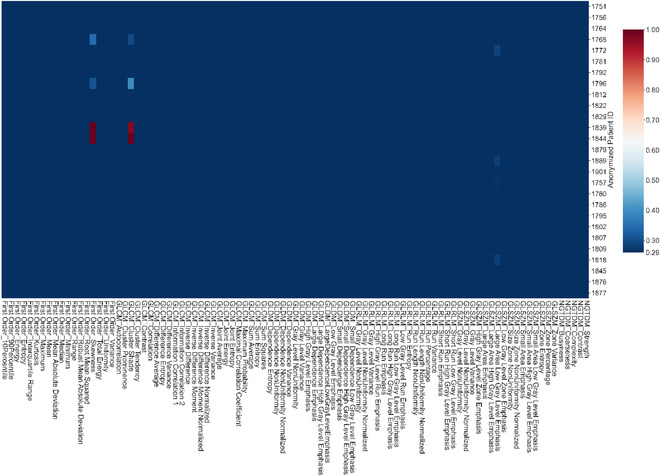
Heatmap showing the coefficient of variation for dosiomic features of rectum. Overall, there were minimal variations (average CV≤0.26) for all the dosiomic features—robust against variations in dose calculation. Two patients had large CVs for cluster shade and skewness

**FIGURE 6:**
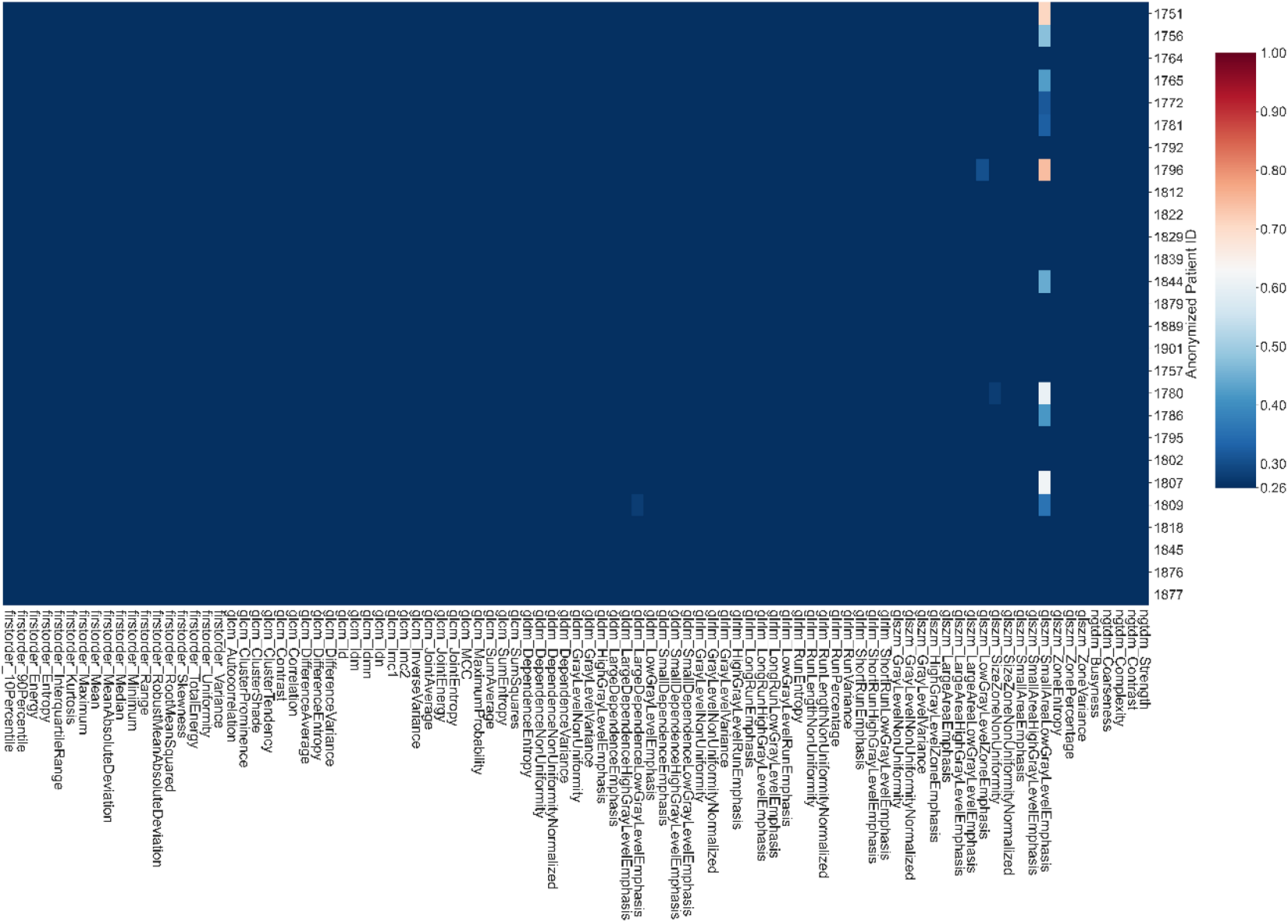
Heatmap showing the coefficient of variation for dosiomic features of bladder. There were minimal variations (average CV≤0.26) for all the dosiomic features except small area low gray level emphasis. The average CV for small area low gray level emphasis was 0.28

The CVs for dosiomic features and DVHs are shown as heatmaps (Figures 2, 3, 4, 5, 6). Each column represents one dosiomic feature (or dosimetric parameter) and each row represents one patient. A dark red cell for a dosiomic feature/dosimetric parameter and a patient means this particular dosiomic feature/dosimetric parameter has a large amount of variation when dose distributions were calculated using different dose calculation algorithm type, version, and dose grid. In the contrary, a dark blue cell means minimal variation, and thus the dosiomic feature/dosimetric parameter is stable against variations in dose calculation details. Among all the ROIs, PTV_High had the greatest range of CVs for the dosiomic features as shown in Figure 2(a). The dosiomic features were clustered into two groups. The green box on the left of Figure 2(a) includes the majority of the features and these features exhibited small CVs. The maximum average CV was 0.26 for large dependency high gray level emphasis. This would be the threshold value we use to decide if a feature is stable or not against variations in dose calculation algorithm type, version, and dose grid.

The purple box on the right of Figure 2(a) includes the six features with large CV (CV > 0.26). They were first‐order feature skewness, GLCM feature cluster shade, and GLSZM features including large area emphasis, large area high gray level emphasis, large area low gray level emphasis, and zone variance. First‐order skewness measures the asymmetry of the voxel intensity distribution of values about the mean value. Cluster shade measures the skewness and uniformity of the gray level co‐occurrence matrix. Large area emphasis measures the distribution of large area size zones, with a greater value indicative of larger size zones and more coarse textures. Large area high gray level emphasis is a measure of the proportion of larger size zones with higher gray level values in the dose distribution. Large area low gray level emphasis is a measure of the proportion of larger size zones with lower gray level values in the dose distribution. Zone variance measures the variance in zone size volumes for all the zones.

On the contrary, in Figure 2(b), the DVHs had minimum CV for all patients and all dosimetric parameters, and they were not detecting as much of variation as dosiomic features in Figure 2(a). The maximum “average CV” was 0.013 for D100%[%], and the second largest “average CV” was 0.006 for D1%[%]. The 12 DVHs for the patient who had the greatest variations in the unstable dosiomic features (patient #1786) are shown in Figure 2(c).

Figures 3, 4, 5, 6 show the results for PTV_Low (Figure 3), PTV_Ring (Figure 4), rectum (Figure 5), and bladder (Figure 6). Although a few patients may have larger variations for certain dosiomic features, overall, there were minimal variations for all the dosiomic features with average CVs*≤*0.26, meaning dosiomic features were stable against variations in dose calculation. The only exception was bladder's small area low gray level emphasis (average CV = 0.28). Meanwhile, DVHs also did not detect much variation (CV < 0.05) in the dose distribution (results not shown).

**FIGURE 7:**
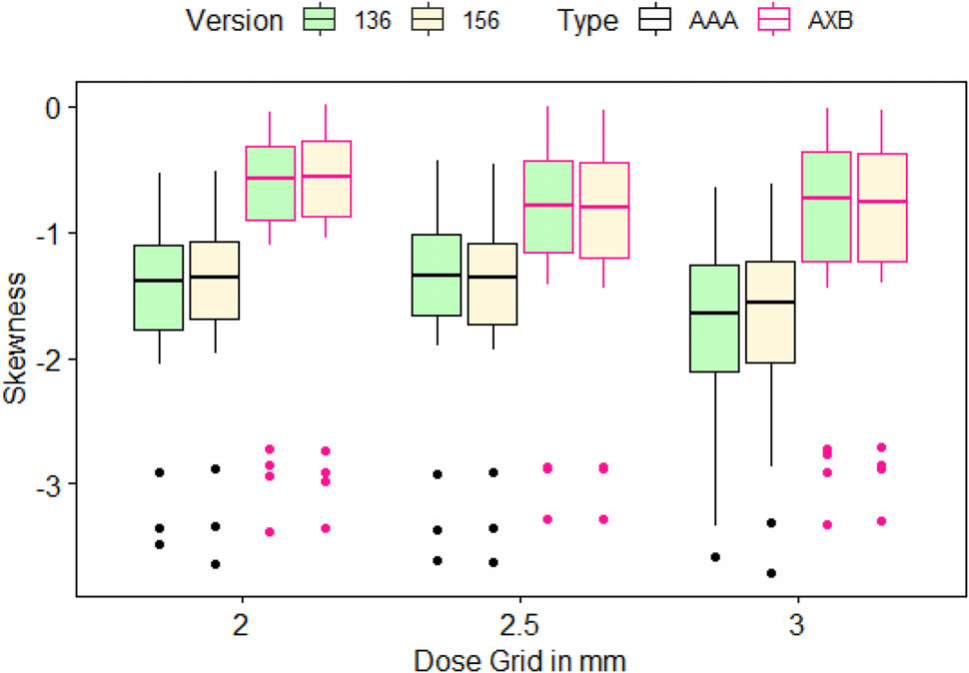
Boxplots showing the variation of PTV_High's skewness due to changes in dose calculation algorithm (DCA) type, version, and dose grid. The dots are the outliers in the original scale

For PTV_High's dosiomic features that showed large variations (average CV > 0.26), the boxplots in Figure 7 and supplementary material Figure S1 visually displayed their variation due to changes in dose calculation algorithm types, versions, and dose grids. The results from three‐way repeated measures ANOVA are summarized in Table 2. The generalized effect size (GES) describes the proportion of the variation in a dependent variable that is due to an effect (e.g., an independent variable). Mathematically, GES is defined as $GES\;=\;\frac{SS_{between\;goups\;}}{SS_{total}},\;$and the groups are classified by the variable of interest.

**TABLE 2:** Results of three‐way repeated measures ANOVA for PTV_High's unstable features

LARGE AREA HIGH GRAY LEVEL EMPHASIS							
	Effect	DFn	DFd	F	*P*		GES
1	Type	1	23	49.3	3.7E‐07	*	10.3%
2	Version	1	23	25.9	3.8E‐05	*	0.1%
3	Dose Grid	1.4	31.9	55.5	1.2E‐09	*	3.5%
4	Type:Version	1	23	0.5	4.8E‐01		0.0%
5	Type:Dose Grid	2	46	23.3	1.0E‐07	*	1.2%
6	Version:Dose Grid	2	46	25.9	2.9E‐08	*	0.1%
7	Type:Version:Dose Grid	2	46	5.0	1.1E‐02		0.0%

LARGE AREA LOW GRAY LEVEL EMPHASIS							
	Effect	DFn	DFd	F	*P*		GES
1	Type	1	24	11.8	2.0E‐03	*	2.6%
2	Version	1	24	28.0	2.0E‐05	*	0.1%
3	Dose Grid	1.3	30.6	44.1	3.5E‐08	*	3.8%
4	Type:Version	1	24	1.6	2.2E‐01		0.0%
5	Type:Dose Grid	2	48	15.8	5.5E‐06	*	1.0%
6	Version:Dose Grid	2	48	18.9	8.6E‐07	*	0.1%
7	Type:Version:Dose Grid	1.6	37.5	3.4	5.3E‐02		0.0%

LARGE AREA EMPHASIS							
	Effect	DFn	DFd	F	*P*		GES
1	Type	1	24	33.4	5.9E‐06	*	6.0%
2	Version	1	24	28.9	1.6E‐05	*	0.1%
3	Dose Grid	1.3	30.6	47.9	1.5E‐08	*	3.7%
4	Type:Version	1	24	1.5	2.3E‐01		0.0%
5	Type:Dose Grid	2	48	18.5	1.1E‐06	*	1.1%
6	Version:Dose Grid	2	48	24.3	5.1E‐08	*	0.1%
7	Type:Version:Dose Grid	1.6	38.6	4.7	2.1E‐02		0.0%

ZONE VARIANCE							
	Effect	DFn	DFd	F	*P*		GES
1	Type	1	24	33.3	6.0E‐06	*	6.0%
2	Version	1	24	28.9	1.6E‐05	*	0.1%
3	Dose Grid	1.3	30.5	47.6	1.7E‐08	*	3.7%
4	Type:Version	1	24	1.5	2.3E‐01		0.0%
5	Type:Dose Grid	2	48	18.5	1.1E‐06	*	1.1%
6	Version:Dose Grid	2	48	24.3	5.1E‐08	*	0.1%
7	Type:Version:Dose Grid	1.6	38.5	4.6	2.2E‐02		0.0%

CLUSTER SHADE							
	Effect	DFn	DFd	F	*P*		GES
1	Type	1	24	52.5	1.7E‐07	*	27.0%
2	Version	1	24	45.6	5.5E‐07	*	0.1%
3	Dose Grid	1.4	33.6	0.7	4.5E‐01		0.1%
4	Type:Version	1	24	2.2	1.5E‐01		0.0%
5	Type:Dose Grid	2	48	4.5	1.6E‐02		0.3%
6	Version:Dose Grid	1.6	37.8	5.0	1.7E‐02		0.0%
7	Type:Version:Dose Grid	2	48	11.4	9.0E‐05		0.0%

SKEWNESS							
	Effect	DFn	DFd	F	*P*		GES
1	Type	1	22	131.1	9.8E‐11	*	47.8%
2	Version	1	22	7.5	1.2E‐02		0.0%
3	Dose Grid	1.2	25.6	29.9	4.8E‐06	*	2.7%
4	Type:Version	1	22	17.8	3.5E‐04	*	0.0%
5	Type:Dose Grid	2	44	25.7	4.1E‐08	*	2.8%
6	Version:Dose Grid	1.3	28.6	39.9	1.2E‐07	*	0.1%
7	Type:Version:Dose Grid	1.3	28.5	24.7	7.5E‐06	*	0.1%

DFn: degree of freedom in the numerator.

DFd, Degree of freedom in the denominator.

Statistically significant: *P* ≤ 0.05/7 = 7.1E‐3, denoted with *.

GES, generalized effect size (the amount of variation in the dosiomic feature due to the corresponding effect).

As shown in Table 2, dose calculation algorithm type, version, and grid size all had a statistically significant impact on unstable GLSZM features including large area high gray level emphasis, large area low gray level emphasis, large area emphasis, and zone variance. dose calculation algorithm type (AAA vs. AXB) had the greatest contribution (GES = 10.3%) to variation in large area high gray level emphasis compared to dose grid size (2.0 mm vs. 2.5 mm vs. 3.0 mm, GES = 3.5%) and version (13.6 vs. 15.6, GES = 0.1%). Dose calculation type (GES = 2.6%) and grid size (GES = 3.8%) had greater contribution to variation in large area low gray level emphasis compared to version (GES = 0.1%). Dose calculation type (GES = 6.0%) and grid size (GES = 3.7%) had greater contribution to variation in large area emphasis compared to version (GES = 0.1%). Dose calculation type (GES = 6.0%) and grid size (GES = 3.7%) had greater contribution to variation in zone variance compared to version (GES = 0.1%). For PTV_High's dosiomic feature cluster shade, only dose calculation algorithm type and version had a statistically significant impact on cluster shade, and dose calculation algorithm type had much greater contribution (GES = 27.0%) to variation in cluster shade than version (GES = 0.1%). For PTV_High's dosiomic feature skewness, only dose calculation type and dose grid size had a statistically significant impact, and algorithm type had much greater contribution (GES = 47.8%) to variation in skewness than dose grid (GES = 2.7%).

**FIGURE 8:**
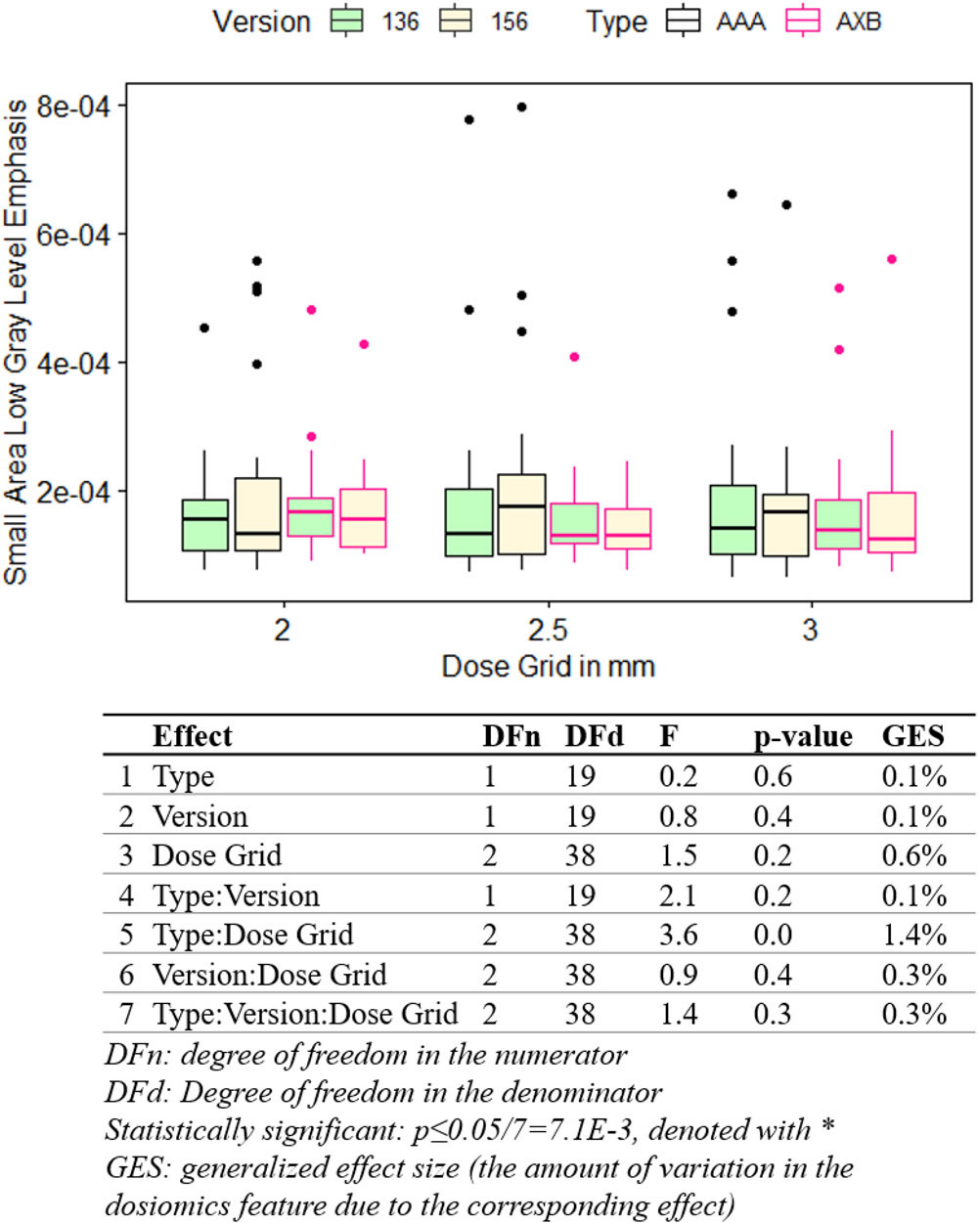
Boxplots showing the variation of bladder's small area low gray level emphasis due to changes in dose calculation algorithm type, version, and dose grid. The dots are the outliers in the original scale. Table showing results of three‐way repeated measures ANOVA. None of the variables had a statistically significant impact on bladder's small area low gray level emphasis

Figure 8 shows the variation in bladder's small area low gray level emphasis due to changes in dose calculation grids, types, and versions, and the results from three‐way repeated measures ANOVA. None of the variables had a statistically significant impact on bladder's small area low gray level emphasis. Table 3 summarizes the major results for dosiomic features in this study.

**TABLE 3:** Summary of the results for dosiomic features in this study

	Version (13.6 vs. 15.6)	Type (AAA vs. AXB)	Dose grid (2 vs. 2.5 vs. 3 mm)	Conclusion^b^
**PTV_Low**	√	√	√	Mixed dose calculation algorithm types, versions, and dose grids acceptable
**PTV Ring**	√	√	√	
**Rectum**	√	√	√	
**Bladder**	None of the three variables led to a statistically significant difference in small area low gray level emphasis			Small area low gray level emphasis had great variation, thus may only be included if dose recalculation is performed
**PTV_High**	Statistically significant for five features and accounted for a minimal amount of variation	Statistically significant for all six features and accounted for a large amount of the variation	Statistically significant for five features and accounted for a moderate amount of variation	Depending on the specific case, all six features may only be included if dose recalculation is performed

^a^ Means all dosiomic features are robust.

^b^ Only apply to scenarios tested in this study.

## 4 DISCUSSION

There is growing interest that the assessment of higher‐order spatial patterns in planned dose distributions can be evaluated against various outcome measures such as tumor control outcome and post‐EBRT toxicity outcome.^1^, ^2^, ^3^, ^4^, ^5^, ^6^, ^7^ Recent advances in machine learning will enable improvements in outcome prediction and outcome‐based plan optimization, however, before we get there, it is necessary to understand and quantify the influence of dose calculation details on dosiomics patterns.

In this study, we examined the stability of dosiomic features against variations in dose calculation algorithm type, version, and dose grid for a cohort of prostate EBRT patients and a selection of ROIs. The results are summarized in Table 3. All the dosiomic features were stable against variations in dose calculation for PTV_Low, PTV_Ring, and rectum. For PTV_High, only first‐order feature skewness, GLCM feature cluster shade, GLSZM features large area emphasis, large area high gray level emphasis, large area low gray level emphasis, and zone variance showed great variation (average CV > 0.26). Dose calculation algorithm type (AAA vs. AXB) and dose grid size (2.0  vs. 2.5  vs. 3.0 mm) were the major sources of variation while dose calculation algorithm version (13.6 vs. 15.6) had a small impact. For bladder, small area low gray level emphasis had great variation due to dose calculation variation, however, none of the three variables showed a statistically significant impact, meaning the variation in small area low gray level emphasis was not systematic among patients.

The four large area related GLSZM features displayed a significant amount of variation within the PTV_High when the dose calculation algorithm and dose grid size changed. These features are related to the number of connected voxels that show the same gray level in the volume. The planning process itself generally seeks to enforce uniform doses across the PTV, which would be reflected in the GLSZM features. Moving from AAA to AXB moves from a superposition‐convolution algorithm to a more direct model of radiation transport, and may have introduced small voxel‐to‐voxel differences in dose, resulting in a change in the number of connected voxels with the same gray level within PTV_High. Further, changes in the size of the dose grid necessarily lead to different dose volumes being averaged, which again, may manifest as variation in the GLSZM features in the context of a relatively uniform PTV dose.

As for the high variation in PTV_High's first order skewness, the dose calculation algorithm (AAA vs. AXB) was the dominant contributing factor. In general, the differential DVHs for dose distributions calculated using AAA were more left‐skewed than AXB. Example DVHs are presented in supplementary material Figure S2. This is consistent with results for PTV_tissue shown in Figure 3 of Padmanaban et al.’s work.^21^ The broader distribution with AXB is likely due to the more detailed radiation transport calculations, accounting more accurately for local variations in material composition and density.

Therefore, when we are relating dosiomic features to treatment outcomes, to produce reproducible and generalizable results, those highly variable features may not be included if the abovementioned dose calculation algorithm types and dose grids were used in the dataset. Meanwhile, when we are reporting dosiomics studies, it is important to follow similar guidelines as recommended by the Image Biomarker Standardization Initiative for reporting radiomics features.^35^ Particularly, reporting the dose calculation algorithm type, version, and dose grid size used is necessary and the ROI used for extracting dosiomic features should be clearly defined.

In general, our results agreed with the published studies^16^, ^17^ that claimed variations in dosiomic features did exist due to inconsistency in treatment planning such as dose calculation algorithm type and grid sizes, and more variations were observed for high dose PTV compared to ROI in the high dose gradient region and adjacent OARs. However, there were differences between our study and the published studies in the category of dosiomic features that were more stable than others. For example, GLRLM, GLSZM, and NGTMD features had zero unstable features while 1/18 first order features, 1/24 GLCM features, 4/16 GLSZM features were unstable in our study for PTV_High. The published results showed that GLCM features almost always had the lowest values for all the settings^16^ and first order dosiomic feature family was more stable than GLCM, GLRLM and GLSZM textural features.^17^ This inconsistency may indicate that the stability of a dosiomic feature can be dependent on the tumor site and specific sources of variation in treatment planning examined.

In studies assessing the reproducibility of radiomics or dosiomic features, a threshold value is usually chosen to decide if a feature is reproducible or not. For example, if the CV value for a feature is ≤ 0.3, the feature is considered to be reproducible^16^; however, the threshold value is chosen fairly arbitrarily and there are no specific and evidence‐based reference threshold values. In this study, we chose the threshold value based on hierarchical clustering results for PTV_High's dosiomic features. The maximum average CV (averaged across all patients) for the group of stable features was 0.26, which can be considered as a threshold value for deciding whether a feature is stable or not. This value is close to the 0.3 threshold value used in the literature.

The cumulative DVH is the most commonly used tool in radiation therapy to evaluate 3D dose distributions. We examined its capability of detecting variations in dose distributions due to variations in dose calculation. Minimum variations in DVHs were found for all the ROIs examined in this study. While some dosiomic features were able to detect significant dose distribution variations in PTV_High, the associated DVHs had minimal CVs by comparison, as demonstrated in Figure 2. This may be due to the fact that DVHs do not contain spatial information and two different dose distributions can have similar DVHs.

Although the 27 patients were scanned on two different Philips Big Bore CT scanners, based on the patients’ hierarchical clustering results in Figure 5, no trend was observed in terms of variations in dosiomic features and which CT scanner was used. It is worth mentioning that the two CT scanners had almost identical electron density calibration curves in our study and the same imaging protocol was used for all the patients. We may need to be cautious when patients were scanned using very different CT scanners with different imaging protocols, especially when there are high‐density implants in the patients. As far as we know, there is no evidence showing that dosiomic features are unrelated to the CT scanner used.

While the CV for the rectum dosiomic features was generally quite small, two patients (patients 1839 and 1844) showed particularly large CVs for rectum GLCM cluster shade and first order skewness (Figure 5). For cluster shade, the values extracted from the dose distributions calculated using AXB were significantly larger than using AAA only for those two patients, indicating greater asymmetry about the mean for the gray level co‐occurrence matrix. For skewness, it was because the mean values for skewness were close to zero for those two patients, as the result, the CV, which equaled to the standard deviation divided by the mean, had a large value. Therefore, we need to be cautions when CV has a large value, it may not actually because large variations are present in the feature of interest but because the mean value is very small.

Based on careful visual examination of these two patients, we noticed that for these two patients, a larger portion of the rectum was inside the high dose PTV and low dose PTV compared to other patients. As an example, the dose distribution and contours for patient 1844 are shown in supplementary material Figure S3. Thus, it is very likely that a larger volume of the rectum of those patients had experienced the steepest dose gradient and differences in radiation transportation calculations may influence the shape of this gradient, particularly when electron equilibrium is not achieved. This may lead to the larger variations manifested in rectum's dosiomic features GLCM cluster shade and first order skewness. Our data suggested that in most cases, these differences are very minor and the dosiomic features are stable; however, the results also suggest that rare cases (2 out of 27 patients examined in this study) may occur when a combination of patient anatomy, contour, and the optimized dose distribution can result in larger variations in some of the dosiomic features.

Only limited dose calculation algorithm types, versions, and dose grid with the Varian treatment planning system were tested in this study, but the difference between AXB and AAA should provide a reasonably wider variation in dose distributions than differences between vendors using similar algorithms. In addition, the methodology used in this study can be used for testing other TPS, across multiple TPS, or testing the impact of different Linear accelerator types. In this work, we focused on TPS versions 13.6 and 15.6, which were released relatively close together in time. Some retrospective outcome studies will have datasets that span wider timelines, particularly when the outcome of interest is a long‐term endpoint, and so the long‐term accumulation of differences in dose calculations is limited in this study to only the two versions studied.

We only tested dosiomic features calculated with Pyradiomics, one of the most commonly used radiomics feature calculation packages. There are a variety of radiomics packages available but according to IBS initiative results, radiomics features derived from the 25 radiomics packages can achieve great agreement with good‐to‐excellent reproducibility.^35^ So, we believe our results are very likely to be generalized to other radiomics packages. Interested researchers can also use the method proposed in this study and use PTV_High as the ROI to test other radiomics packages. Additionally, settings related to dosiomic feature extraction such as resampling voxel size and interpolation method can potentially impact the dosiomic features. The approaches and values used in this study are the most commonly used ones in dosiomics studies, but it is worth further investigating on how the specific settings can impact the extracted dosiomic features. As for the dose bin count, there are no other specific guidelines in literature on the exact dose bin count/width that should be used. It is recommended by Pyradiomics to use a bin count in the range of 30 to 130, because it has been shown to have good reproducibility and performance in literature.^36^ There is a heterogeneity in the dose bin count or dose bin width used among the published dosiomics studies that reported values used.^16^, ^17^, ^37^ Further studies are needed to demonstrate the impact of dose bin count/width on the robustness of dosiomics features.

Furthermore, only dosiomic features extracted from the original dose distribution were examined in this study and none of the filters or dosiomic features extracted from the filtered dose distributions were investigated. As more studies start to investigate the prognostic ability of dosiomic features extracted from filtered dose distribution, it is important to investigate the robustness of those features against dose calculation details in the future. A recently published Japanese study has demonstrated that dose calculation algorithms can affect the reproducibility of wavelet filter‐based dosiomic features for lung SBRT.^38^

Another limitation of this study is that only prostate cancer EBRT plans were examined in this study, and results derived in this study may not be generalized to other disease sites. For example, for tumor sites with greater tissue inhomogeneity such as lung, the impact of dose calculation algorithm on the dose distribution can be more significant compared to the pelvic region, which is more homogeneous. Differences in medium heterogeneity, patient anatomy, approaches to optimization, etc. may more strongly influence the distribution of dosiomic features between patients. Interested researchers can follow the workflow in this study and investigate relevant ROIs for other tumor sites.

## 5 CONCLUSION

Dosiomic features for low dose PTV, PTV Ring, and rectum were stable against the variations in dose calculation examined in this study. Most of the high dose PTV's dosiomic features were stable except for six features that had great variations due to dose calculation algorithm type change and dose grid change. All of the bladder's dosiomic features were stable except for one feature. DVHs for all the examined regions of interest showed minimum variation.

## AUTHOR CONTRIBUTION

Lingyue Sun conceived this project, acquired data, performed analysis, wrote the initial draft, and revised the manuscript. Wendy Smith and Charles Kirkby contributed substantially to the design of this project, interpretation of the data, review and revision of the manuscript. All authors approved submission of this manuscript.

## CONFLICT OF INTEREST

No conflicts of interest.

## ETHICAL APPROVAL STATEMENT

This study is approved by the local ethics board, the Health Research Ethics Board of Alberta—Cancer Committee (HREBA.CC‐19‐0329).

## Supporting information

Supporting Information
