# Supplementary material for: Stability of dosiomic features against variations in dose calculation: An analysis based on a cohort of prostate external beam radiotherapy patients
Source: J Appl Clin Med Phys. 2023 Jan 11;24(5):e13904. doi: 10.1002/acm2.13904 (PMC10161028; doi:10.1002/acm2.13904)
Supplement: Supplementary file 1 [file ACM2-24-e13904-s002.docx]

**Supplementary Material 2 - Figures:**


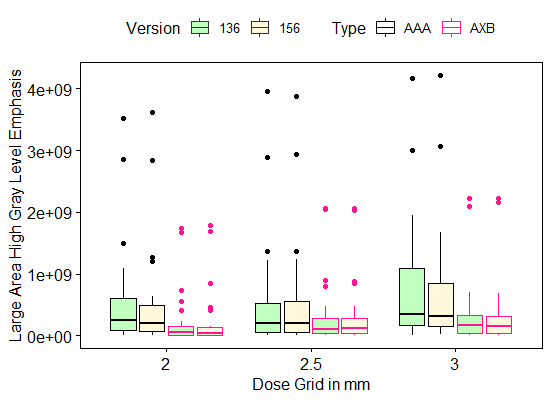


*
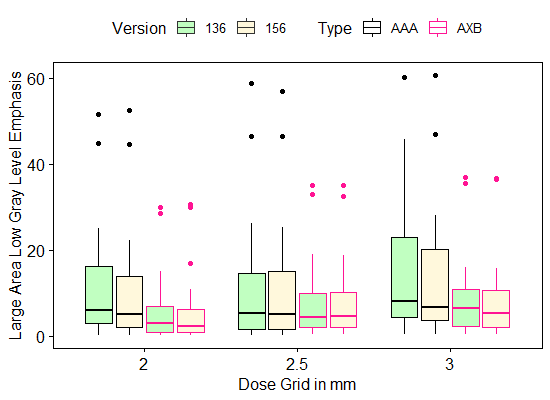
*

*
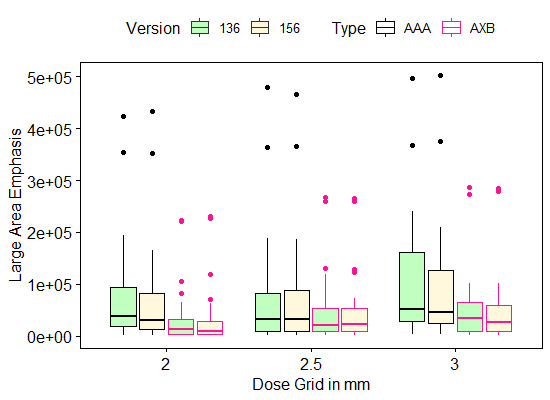
*

*
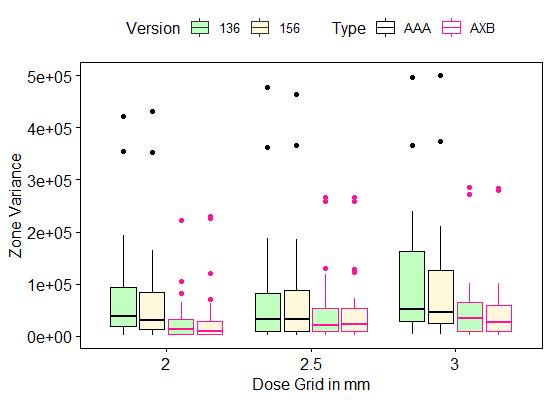
*

*
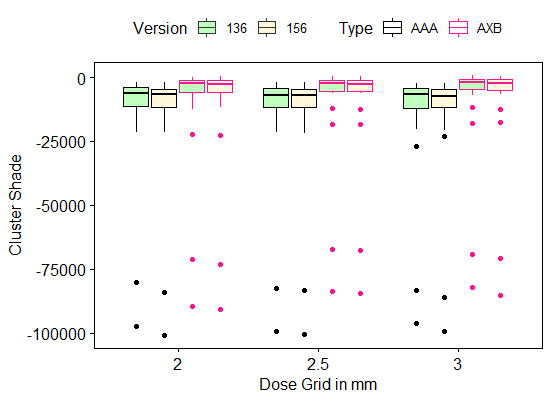
*

*Figure S1: Boxplots showing the variation of PTV_High’s unstable features (large area high gray level emphasis, large area low gray level emphasis, large area emphasis, zone variance, and cluster shade) due to changes in dose calculation algorithm (DCA) type, version, and dose grid. The dots are the outliers.*


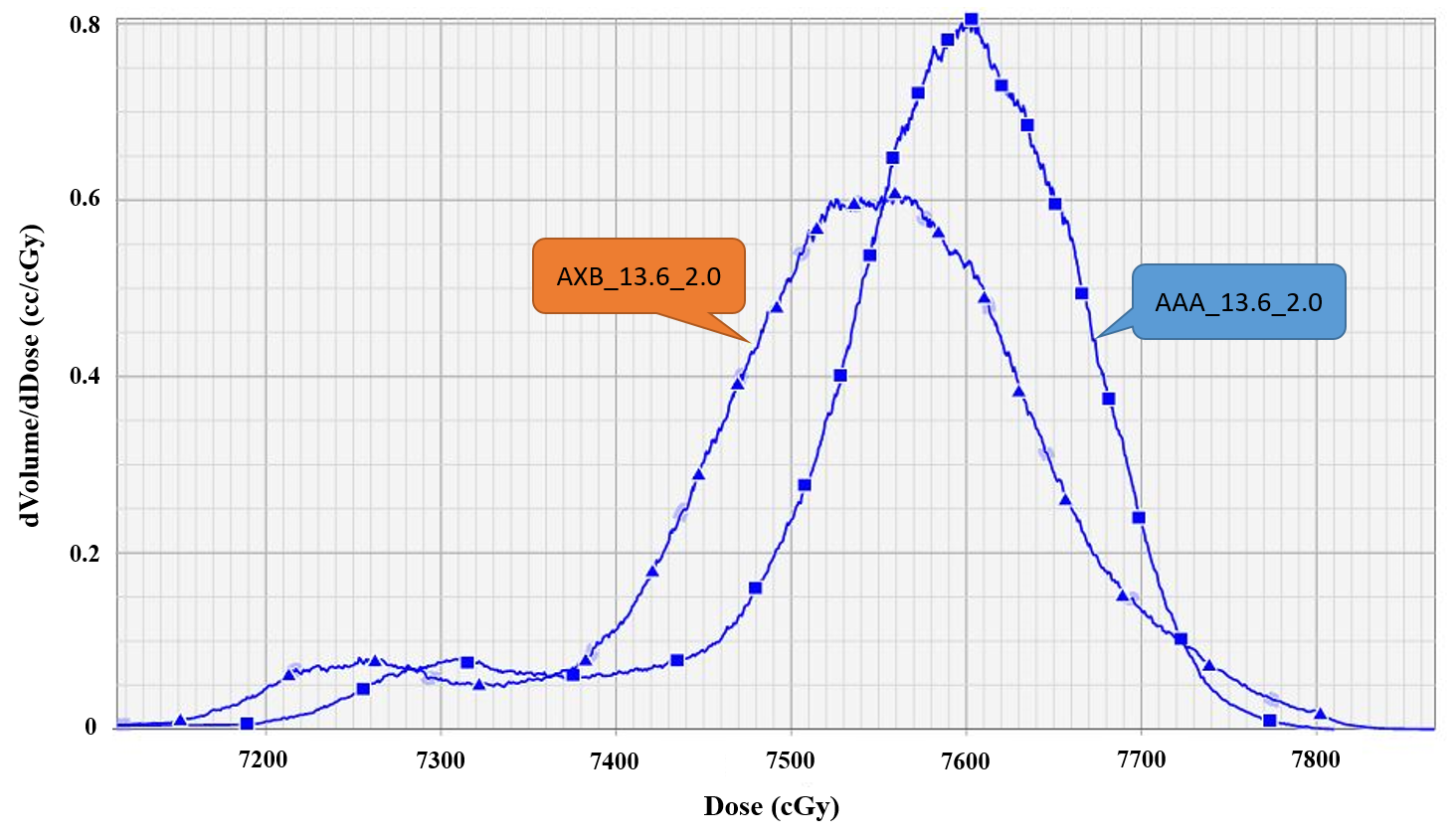


*Figure S2: Differential DVHs for the PTV_High from two dose distributions: one dose distribution was calculated using AXB with a dose grid size of 2.0mm on Eclipse version 13.6, and the other was calculated using AAA with a dose grid size of 2.0mm on Eclipse version 13.6. The differential DVH for AAA was more left-skewed and pointier.*

*
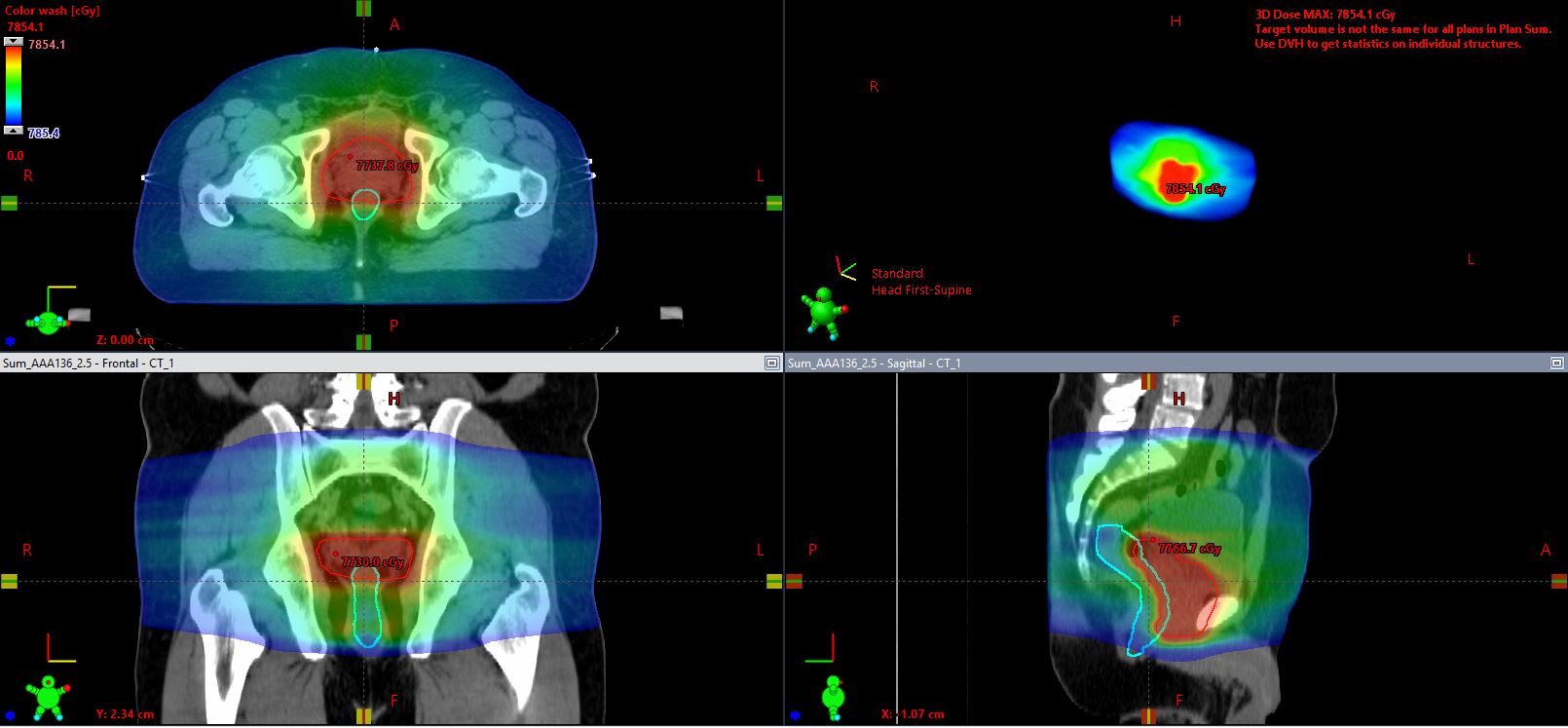
*

*Figure S3: The dose distribution and contours for patient 1844. The cyan contour represents the rectum and the red contour represents the high dose PTV (PTV_High).*
