# Supplementary material for: Stability of dosiomic features against variations in dose calculation: An analysis based on a cohort of prostate external beam radiotherapy patients
Source: J Appl Clin Med Phys. 2023 Jan 11;24(5):e13904. doi: 10.1002/acm2.13904 (PMC10161028; doi:10.1002/acm2.13904)
Supplement: Supplementary file 2 — Supporting Information [file ACM2-24-e13904-s001.docx]

**Supplementary Materials:**

*Table S1. A Brief Description of Each Gray Level Matrix.*

| **Name of Gray Level Matrix** | **Description*** |
| --- | --- |
| Gray Level Co-occurrence Matrix | A gray level co-occurrence matrix represents the second order joint probability function of a dose distribution region, which is defined by a binary mask. The (i, j) element of the matrix represents the number of times the combination of dose level i and j separated by a pre-specified distance and along a specific direction occur in two voxels in the dose distribution. |
| Gray Level Run Length Matrix | A gray level run length matrix quantifies the number of gray level runs in a dose distribution. A gray level run is a series of connected voxels that have the same gray level intensity along a certain direction. The (i, j) element of the matrix represent the number of gray level runs with gray level i and length j in the dose distribution constraint by a binary mask. |
| Gray Level Size Zone Matrix | A gray level size zone matrix quantifies the number of gray level zones in a dose distribution. A gray level zone is a series of connected voxels that have the same gray level intensity. The (i, j) element of the matrix represents the number of gray level zones with gray level i and size j in the dose distribution constraint by a binary mask. |
| Gray Level Dependency Matrix | A gray level dependency matrix quantifies the number of gray level dependencies in a dose distribution. A gray level dependency is a series of voxels within a certain distance that have a pre-specified gray level intensity difference. The (i, j) element of the matrix quantifies the number of times a voxel with gray level i has j dependent voxels. |
| Neighboring Gray-Tone Difference Matrix | A neighbouring gray tone difference matrix presents the gray value difference between a voxel and its neighbours that are a certain distance away. |

* More detailed description can be found in PyRadiomics website: <https://pyradiomics.readthedocs.io/en/latest/features.html>

*Table S2. Dosiomics Features Extracted Using PyRadiomics*

| **First Order Statistics Features**  **(18 features)** | 10^th^ Percentile | **Gray Level Co-occurrence Matrix Features**  **(24 GLCM features)** | Autocorrelation |
| --- | --- | --- | --- |
|  | 90^th^ Percentile |  | Cluster Prominence |
|  | Energy |  | Cluster Shade |
|  | Interquartile Range |  | Cluster Tendency |
|  | Entropy |  | Contrast |
|  | Kurtosis |  | Correlation |
|  | Maximum |  | Difference Average |
|  | Mean Absolute Deviation |  | Difference Entropy |
|  | Mean |  | Difference Variance |
|  | Median |  | Inverse Difference |
|  | Minimum |  | Inverse Difference Moment |
|  | Range |  | Inverse Difference Normalized |
|  | Robust Mean Absolute  Deviation |  | Inverse Difference Moment Normalized |
|  | Root Mean Squared |  | Information Correlation 1 |
|  | Skewness |  | Information Correlation 2 |
|  | Total Energy |  | Inverse Variance |
|  | Uniformity |  | Joint Average |
|  | Variance |  | Joint Energy |
| **Neighboring Gray-Tone Difference Matrix Features**  **(5 NGTDM features)** | Busyness |  | Joint Entropy |
|  | Coarseness |  | Maximum Probability |
|  | Complexity |  | Sum Average |
|  | Contrast |  | Sum Entropy |
|  | Strength |  | Sum Squares |
|  |  |  | Maximal Correlation  Coefficient |
| **Gray Level Run Length Matrix Features**  **(16 GLRLM features)** | Gray Level NonUniformity | **Gray Level Size Zone Matrix Features**  **(16 GLSZM features)** | GrayLevel NonUniformity |
|  | Gray Level NonUniformity Normalized |  | GrayLevel NonUniformity Normalized |
|  | Gray Level Variance |  | Gray Level Variance |
|  | High Gray Level Run Emphasis |  | High Gray Level Zone Emphasis |
|  | Long Run Emphasis |  | Large Area Emphasis |
|  | Long Run High Gray Level  Emphasis |  | Large Area High Gray Level Emphasis |
|  | Long Run Low Gray Level  Emphasis |  | Large Area Low Gray Level Emphasis |
|  | Low Gray Level Run Emphasis |  | Low Gray Level Zone Emphasis |
|  | Run Entropy |  | Size Zone NonUniformity |
|  | Run Length NonUniformity |  | Size Zone NonUniformity Normalized |
|  | Run Length NonUniformity Normalized |  | Small Area Emphasis |
|  | Run Percentage |  | Small Area High Gray Level Emphasis |
|  | Run Variance |  | Small Area Low Gray Level Emphasis |
|  | Short Run Emphasis |  | Zone Entropy |
|  | Short Run HighGray Level Emphasis |  | Zone Percentage |
|  | Short Run Low Gray Level Emphasis |  | Zone Variance |
| **Gray Level Dependency Matrix Features**  **(GLDM 14 features)** | Dependence Entropy |  |  |
|  | Dependence NonUniformity |  |  |
|  | Dependence NonUniformity Normalized |  |  |
|  | Dependence Variance |  |  |
|  | Gray Level NonUniformity |  |  |
|  | Gray Level Variance |  |  |
|  | High Gray Level Emphasis |  |  |
|  | Large Dependence Emphasis |  |  |
|  | Large Dependence High Gray Level Emphasis |  |  |
|  | Large Dependence Low Gray Level Emphasis |  |  |
|  | Low Gray Level Emphasis |  |  |
|  | Small Dependence Emphasis |  |  |
|  | Small Dependence High Gray Level Emphasis |  |  |
|  | Small Dependence Low Gray Level Emphasis |  |  |
